# Advances and Challenges in Vaccine Development for West Nile Virus (WNV) Infection

**DOI:** 10.3390/vaccines14060499

**Published:** 2026-06-02

**Authors:** Anjali Gupta, Aarti Tripathi, Kirtika Jha, Yogita Rawat, Urvashi Bhardwaj, Renu Khasa, Shailendra Chauhan

**Affiliations:** 1 New Jersey Medical School, Rutgers University, Newark, NJ 07103, USA; 2 Department of Pathology, University of Texas Medical Branch, Galveston, TX 77555, USA; 3 Galveston National Laboratory, Galveston, TX 77555, USA; 4 Tata Institute for Genetics and Society, Bengaluru 560065, India; 5 Department of Biochemistry, School of Chemical and Life Sciences, Jamia Hamdard University, New Delhi 110062, India; 6 Department of Cellular and Molecular Medicine, Herbert Wertheim College of Medicine, Florida International University, Miami, FL 33199, USA

**Keywords:** West Nile Virus, vaccine, bird vaccine, equine vaccine, human vaccine

## Abstract

West Nile Virus (WNV) belongs to the orthoflavivirus genus and is part of the Flaviviridae family, which includes the Japanese encephalitis virus, Dengue virus, Zika virus, and yellow fever virus. WNV circulates among birds and mosquitoes, posing infection risks to humans and mammals. The significant rise in WNV’s geographic spread and infection rates over the past five decades has prompted urgent public health concerns, driving the need for accelerated vaccine research. The development of a vaccine for WNV infection presents several challenges, primarily due to the virus’s complex biology, the risk of cross-reactivity with other flaviviruses, safety concerns such as antibody-dependent enhancement (ADE), and the economic and logistical hurdles in vaccine production. Despite significant research efforts, no human vaccine has been approved, although several candidates are in various stages of development. The current review offers a comprehensive summary of the latest progress and the concomitant challenges in the development of vaccines. It also discusses the role of host–pathogen interaction, host immunity, viral immune evasion, and disease pathogenesis in facilitating the advancement of vaccines.

## 1. Introduction

WNV is a flavivirus that is spread by mosquitoes. It was first found in Uganda in 1937. It has since become a major global public health issue because it is now widespread and can cause severe neuroinvasive disease in both humans and animals. After the virus was introduced into North America in 1999 [1], it rapidly spread and became endemic, contributing to repeated seasonal outbreaks across multiple continents. These outbreaks continue to create substantial health and economic pressures on affected regions. Although extensive research efforts have been made over the past two decades, there is still no licensed vaccine available for human use, even though several candidates have demonstrated promising results in preclinical studies and early clinical trials [2]. The development of a safe and effective WNV vaccine has been slowed by scientific, regulatory, and economic barriers. One major challenge is the complex immunopathogenesis of WNV, which involves both protective and potentially harmful immune responses [3]. The development of vaccines must balance minimizing safety issues like ADE, which has been observed in some related flaviviruses, with achieving high protective immunity. Cross-reactive immune responses among flaviviruses, including Dengue, Zika, and Japanese encephalitis viruses, further complicate vaccine research [4]. This review discusses the wide range of challenges that have slowed progress in developing a human vaccine for WNV, along with the vaccines available for birds and equines. Earlier studies have found that progress in this area is still slowed by several challenges. These include the host’s complex immune responses, the relatively small market demand for certain vaccines, and technical difficulties involved in designing them. By examining current vaccine strategies and identifying the main gaps that remain, this review aims to highlight the directions for future research that could help to produce a safe and effective WNV vaccine for human use.

## 2. WNV Structure, Replication, Geographical Distribution, Diagnosis and Treatment

### 2.1. WNV Genome

WNV is an enveloped virus with a single-stranded, positive-sense RNA genome. The genome is approximately 11 kb in length and contains a single open reading frame without a polyadenylated tail at the 3′ end. Highly conserved stem loop structures located at both the 5′ and 3′ untranslated regions play essential roles in viral replication, translation, transcription, and genome packaging [5,6,7].

**Figure 1:**
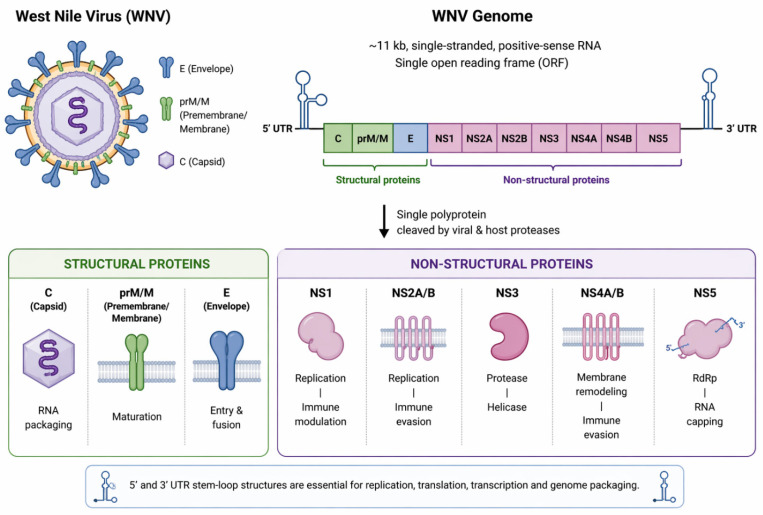
WNV and genome structure.

The viral genome encodes three structural proteins: capsid (C), premembrane/membrane (prM/M) and envelope (E) along with seven non-structural proteins including NS1, NS2A, NS2B, NS3, NS4A, NS4B, and NS5. These proteins are initially synthesized as a single polyprotein, which is subsequently cleaved co-translationally by both viral and host proteases. The structural proteins encoded at the 5′ end of the genome are critical for viral attachment, membrane fusion, entry into host cells and encapsidation of the viral RNA during virion assembly [8]. The limited coding capacity of the viral genome necessitates that non-structural proteins perform multiple functions during infection. Among these, NS1 exists in both intracellular and secreted forms and is also highly immunogenic. Although NS1 is not directly involved in virion assembly, exsting evidence suggests that it contributes to viral replication and may affect host immune responses [9]. NS3 is the viral protease that cleaves non-structural proteins from the viral polyprotein and encodes enzyme activities, functions that have been widely characterized [10,11]. NS5 functions as the viral RNA-dependent RNA polymerase and also contains a methyltransferase domain. Both of these are essential for viral genome replication and RNA capping [8,12]. In addition, several non-structural proteins including NS2A, NS2B, NS4A, and NS4B contribute to immune evasion by interfering with key components of the host innate antiviral response [13,14,15,16] (Figure 1).

### 2.2. WNV Replication

WNV can replicate in various cell types from many species, including mammals, birds, amphibians, and insects. The virus enters cells by binding its E protein to specific receptors [17]. Key co-receptors include DC-SIGN and DC-SIGN-R in dendritic cells [18], as well as integrin αvβ3, which interacts with the RGD/RGE sequence in domain III of the E protein [19]. However, some cell types do not require integrin αvβ3 for entry, indicating that receptor usage may vary by strain and cell type [20]. WNV can sneak into cells even without a normal entry receptor by displaying phosphatidylserine (PS) on its surface, making it look like a dying cell. Because of this disguise, the cell mistakes the virus for harmless debris and pulls it inside using PS-recognition systems like the TIM and TAM protein families. This clever “apoptotic mimicry” allows the virus to enter through pathways such as macropinocytosis or clathrin-mediated endocytosis, where it then fuses inside the acidic endosome. By posing as something the body should clear away rather than fight, WNV is able to infect many different cell types and avoid early immune detection [21,22,23]. Rab5 GTPase is essential for the entry of both WNV and Dengue virus [24]. The laminin-binding protein has also been identified as a specific receptor for WNV, with high efficiency in binding to domain II of the E protein [25,26]. Following entry into the host cell, WNV releases its RNA genome through a tightly regulated process. After endocytosis, acidification of the endosome induces a conformational change in the viral envelope (E) protein, enabling fusion of the viral and endosomal membrane. This fusion event releases the nucleocapsid into the cytoplasm. The genomic RNA dissociates from the capsid and becomes immediately available for translation by host ribosome thereby initiating the viral replication cycle [27]. The resulting viral polyprotein is subsequently cleaved by the viral NS3 serine protease and host signal peptidases within the endoplasmic reticulum. Concurrently, the viral RNA-dependent RNA polymerase synthesizes complementary negative-sense RNA intermediates from the positive-sense genomic RNA. These negative-sense strands serve as templates to produce multiple new positive-sense genomic RNAs. Notably, RNA replication can occur independently of ongoing protein synthesis. Typically, a single positive-sense RNA template gives rise to only one negative-sense strand at a time. Each negative-sense intermediate can generate multiple positive-sense RNA molecules simultaneously [28,29]. In contrast, virion assembly is highly dependent on adequate protein synthesis. Each mature virion requires one copy of genomic RNA along with 180 copies each of the E and prM structural proteins for proper particle formation.

WNV builds new viral particles inside the endoplasmic reticulum, where they first form as immature, non-infectious virions. These particles travel through the cell’s normal secretory pathway moving from the ER to the Golgi like ordinary cargo. Inside the acidic trans-Golgi network, a host enzyme called furin cuts the prM protein, reshaping the particle into a fully infectious virus. The mature virions are then packed into vesicles and carried toward the surface of the cell. Finally, the vesicles fuse with the plasma membrane and release the virus by exocytosis, allowing infection to continue without killing the host cell [27,30,31].

### 2.3. Transmission Cycle

**Figure 2:**
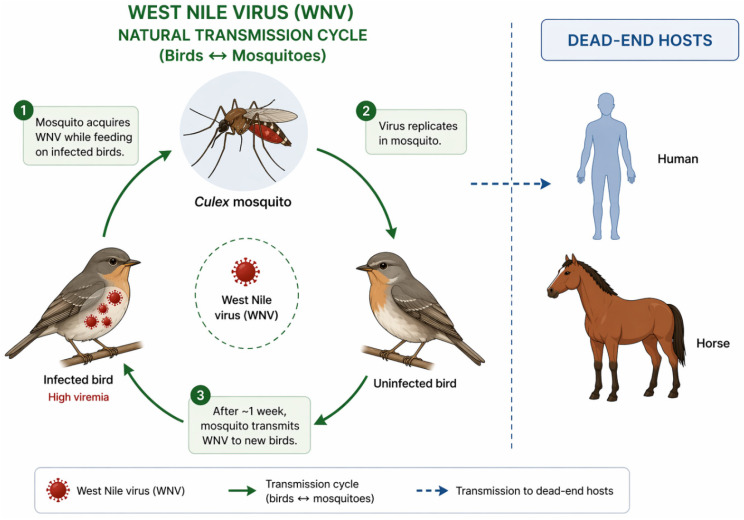
Virus transmission cycle.

WNV is most commonly transmitted to humans through the bite of an infected mosquito. Mosquitoes acquire the virus when they feed on infected birds which serve as the primary amplifying hosts. The natural transmission cycle of the virus is therefore maintained predominantly between birds and mosquitoes especially species belonging to the genus *Culex* [32,33,34]. Infected birds often develop high levels of viremia allowing feeding mosquitoes to readily acquire the virus and perpetuate the transmission cycle. Around a week later, these mosquitoes can transmit the virus to more birds. Mosquitoes can also infect humans, horses, and other mammals which are the “dead-end” host [35] (Figure 2).

### 2.4. Geographical Distribution

The first recognized WNV epidemic occurred in Haifa, Israel, in 1951, presenting symptoms like febrile illness, rash, lymphadenopathy, and angina [36]. At the same time, WNV was isolated from febrile children and *Culex* mosquitoes in Egypt [37]. In 1953, the virus was found in hooded crows and rock pigeons [38]. During the 1957 outbreak in Israel, neurological symptoms were seen in 33 percent of patients, with a four percent mortality rate among elderly nursing home residents [39]. Subsequent outbreaks followed in France and South Africa in the 1960s and 1970s. The virus spread to Europe, China, and Australia, and the first human case in South America was reported in Colombia in 2005, with Argentina recording its first encephalitis case in 2006 [40]. From 2015 to 2016, WNV neuroinvasive disease cases were noted in Sindh, Pakistan, while protective antibodies in the population explained the delayed emergence of these cases. Sri Lanka recorded its first WNV infection in 2013 [41]. In India, antibodies against West Nile virus were first detected in human serum samples collected in Mumbai in 1952 indicating that the virus has been circulating in the country for several decades. Since then, multiple reports have documented severe neuroinvasive diseases associated with WNV particularly among children. In 1980 and 1981, the virus was isolated from the brain tissue of three children who died from encephalitis in southern India.

In addition to encephalitis, WNV has also been associated with acute flaccid paralysis (AFP), an uncommon but important neurological manifestation. In Kerala, laboratory-confirmed cases of WNV-associated AFP were reported in 2014 [42]. This finding was notable because AFP in India has historically been most strongly associated with poliovirus infection. Molecular characterization of WNV positive samples collected during the 2011 outbreak in Kerala demonstrated the circulation of lineage I strains. Similar lineage I infections were later identified among pediatric patients with acute encephalitis syndrome in Madhya Pradesh in 2015. Subsequent reports also documented WNV activity in West Bengal in 2017 suggesting an expanding geographic distribution of the virus within India [43].

### 2.5. Disease Manifestation

WNV infection can range from a self-limiting febrile illness to severe neurological disease including meningitis, encephalitis and acute flaccid myelitis [44]. The incubation period is typically 2–6 days, although it may range from 2 to 14 days and can be longer in immunocompromised individuals [45]. Among patients with neuroinvasive disease, the overall case fatality rate is approximately 10% and survivors frequently experience persistent neurological sequelae particularly after encephalitis or acute flaccid myelitis [46,47].

WNV meningitis is clinically similar to other forms of viral meningitis and commonly presents with fever, headache and nuchal rigidity [48]. In contrast, WNV encephalitis is generally more severe and is characterized by fever, altered mental status, seizures, focal neurological deficits and movement disorders such as tremor or parkinsonian features [49].

Acute flaccid myelitis associated with WNV closely resembles poliomyelitis both clinically and pathologically. The disease primarily affects anterior horn cells in the spinal cord, resulting in asymmetric limb weakness or paralysis. In severe cases, respiratory muscle involvement can lead to respiratory failure requiring mechanical ventilation [50,51]. Notably, WNV-associated acute flaccid myelitis may occur in the absence of fever or other overt viral symptoms. In addition, WNV infection has also been linked to neurological complications such as Guillain–Barré syndrome and radiculopathy which can usually be distinguished from acute flaccid myelitis based on their distinct clinical features [48].

### 2.6. Diagnosis and Treatment

Diagnosis of WNV focuses on identifying either the immune response to infection or the virus itself. The most widely used approach is detecting WNV-specific IgM antibodies in serum or cerebrospinal fluid (CSF), which typically appear within the first few days of illness and indicate a recent infection. Because IgM can persist for months, results are often confirmed using plaque-reduction neutralization tests (PRNTs), which help distinguish WNV from other flaviviruses that may cause false-positive results. In patients with neurological symptoms, IgM in CSF is particularly valuable because it normally does not cross the blood–brain barrier, so its presence strongly supports neuroinvasive disease. Direct viral detection methods, such as RT-PCR, can identify WNV RNA in blood or CSF early in infection, although sensitivity decreases as viremia declines after antibody production begins. In severe or fatal cases, immunohistochemistry or PCR on tissue samples can also be used to confirm infection. Because clinical symptoms alone are not reliable for diagnosis, combining serology, molecular testing, and PRNT confirmation when needed provides the most accurate results. Timely and reliable WNV diagnosis not only guides patient management but also supports outbreak monitoring and public health interventions [52,53,54].

The current therapeutic approach for WNV involves several treatments, including corticosteroids, immune γ-globulin, monoclonal antibodies, interferon α-2b, antisense oligomers, and anticonvulsants or osmotic agents [55,56]. However, there have been no controlled studies to establish the therapeutic efficacy of these treatments [55]. Various antiviral agents have been investigated either in vitro using WNV-infected cell lines or in vivo with laboratory animals. Additionally, natural compounds such as isoflavones have shown strong antiviral activity against a range of RNA viruses [57]. Some antibiotics have demonstrated anti-flavivirus activities in vitro [58]. Interestingly, an experimental study found that administering oral doses of vancomycin, neomycin, ampicillin, and metronidazole in mice worsened the disease severity associated with multiple flavivirus infections [59].

At present, there are no licensed vaccines or specific antiviral treatments available for human infection with West Nile virus. Several vaccines have been developed for equine use. These include three inactivated whole-virus vaccines: WN Innovator, Vetera WNV and Prestige WNV as well as one live chimeric vaccine, Recombitek Equine WNV. Most of these vaccines are derived from the NY99 strain with the exception of Vetera WNV, which is based on a different viral isolate. Collectively, these vaccines provide protective immunity in horses for approximately one year [60].

Several vaccine candidates for human use are currently under preclinical development, and a few have advanced to phase II clinical trials. However, none of them have progressed to phase III efficacy studies. This is largely due to the sporadic and unpredictable nature of WNV outbreaks which makes large-scale efficacy trials difficult to conduct as well as concerns regarding the limited commercial market for such vaccines [55,61,62,63]. Additionally, humans are dead-end hosts for the virus, raising questions about the cost-effectiveness of a human vaccine [2]. Researchers are also developing a vaccine targeting mosquito saliva proteins, which could protect against multiple mosquito-borne pathogens [55,64].

### 2.7. Preventive Measures

At present, prevention remains the most effective strategy for reducing the burden of West Nile virus infection as no licensed human vaccine or specific antiviral therapy is available. Public health efforts rely heavily on surveillance, early warning systems and vector control measures. The development of comprehensive monitoring programs for mosquito populations, bird reservoirs, and human cases is essential for identifying outbreaks early and limiting viral transmission to humans [65]. Effective surveillance programs for WNV mosquito vectors are crucial because they help detect when viral activity is increasing long before people start getting sick. By regularly monitoring mosquito populations, virus infection rates, and bird mortality, public health teams can identify hotspots where transmission is likely to intensify. This early warning system allows authorities to respond proactively rather than reactively by increasing mosquito control measures, alerting communities, and preparing healthcare services. Surveillance also shows whether prevention strategies are working or need adjustment, rather than waiting for human cases to appear. Overall, strong vector surveillance protects communities by turning silent environmental signals into timely action that reduces the risk of a human outbreak.

## 3. WNV Vaccine Development and Challenges in Birds

WNV circulates in a wide range of hosts, including mammals and birds; however, their host competence in virus transmission differs substantially. Mammals such as humans and horses, when exposed to WNV, may develop severe diseases, but they typically generate low and transient viremia that is insufficient to infect mosquitoes, thus serving as dead-end hosts [66]. In contrast, birds play a pivotal role in the natural transmission cycle of WNV, as they develop high and sustained viremia following infection, allowing efficient transmission to mosquitoes. Among birds, members of the order Passeriformes, especially family Corvidae, including crows, jays, and magpies, are particularly susceptible and serve as potent amplifying hosts. These species often develop extremely high viral titers in blood [67]. Experimental studies have shown that viremia in birds can be detected as early as one day post-infection and may persist for 7–11 days, a window during which mosquitoes readily acquire the virus [68].

### 3.1. Ecological Impact on Birds and Disease Severity

Since the introduction of WNV into the United States in 1999, virus-associated morbidity and mortality have been documented in nearly 300 avian species, including endangered species and synanthropic birds that live in proximity to humans. Corvid populations, in particular, experienced dramatic declines during early outbreaks, underscoring their importance in WNV epidemiology and surveillance [1]. In addition to mosquito-mediated transmission, birds can also contract WNV by oral exposure, ingestion of infected prey, and direct bird-to-bird contact. Viral shedding via oral and fecal routes promotes transmission in communal roosting and nesting environments. Both wild and domestic birds can develop severe diseases following WNV infection. The virus disseminates to multiple organs, including the liver, spleen, kidneys, heart, and central nervous system. Neuro invasion of the virus may result in encephalitis, neurological dysfunction, and death, often occurring within 24–48 h in highly susceptible species [68].

### 3.2. Immune Responses and Rationale for Avian Vaccination

Infected birds that survive WNV infection typically develop long-lasting neutralizing antibodies, and maternal antibodies can be transferred to chicks. These findings have encouraged efforts to develop vaccines aimed at reducing avian viremia and disrupting the mosquito–bird transmission cycle [69]. Several vaccination platforms have been evaluated in birds, including inactivated whole-virus vaccines, live attenuated vaccines, recombinant subunit vaccines, chimeric viral vaccines, and DNA-based vaccines. Many of these candidates were initially developed for equine use and later adapted for avian studies [69,70].

### 3.3. Performance of Equine-Derived Vaccines in Birds

Formalin-inactivated WNV vaccines originally developed for horses have been tested in birds such as geese. In controlled studies, laboratory-vaccinated geese showed approximately 86.6% protection, while farm-vaccinated birds achieved about 75.3% protection. Although these vaccines reduced mortality and disease severity, their efficacy has not yet been widely tested across diverse avian species [70].

### 3.4. DNA and Recombinant Vaccine Approaches

Initially licensed for horses in 2004 as West Nile-Innovator, a DNA vaccine by Fort Dodge was evaluated for safety and effectiveness in falcons. Vaccinated falcons developed immunity more quickly, had shorter viremia, and shed less virus than unvaccinated controls after WNV exposure. Additionally, vaccinated birds displayed milder brain lesions, suggesting protection against WNV-induced encephalitis. With no observed adverse effects and reduced infection severity, this vaccine is recommended for falcons, while studies continue to evaluate its use in chicks [71]. DNA vaccines encoding WNV prM and E proteins, including constructs such as pCBWN and plasmids developed by Aldevron, have demonstrated immunogenicity in birds. These vaccines reduce viremia and disease severity, although complete protection has not been consistently achieved [72].

A recombinant subunit protein vaccine produced in genetically modified HeLa-3 cells showed promising results in magpies, a natural WNV amplifier host. A single intramuscular dose significantly reduced viremia, decreased viral shedding from feathers, and improved survival rates to approximately 71.4%. The findings suggest that this vaccine could be used effectively and safely to limit the WNV spread in avians, particularly in communal bird populations like magpies and crows [73] (Table 1). The chimeric ChimeriVax-WN vaccine, based on the yellow fever 17D backbone, was tested in fish crows. Intramuscular administration provided partial protection and reduced viremia to levels less likely to infect mosquitoes, whereas oral vaccination was ineffective. The limited durability of the immune response highlights the need for booster strategies [74]. Birds’ WNV vaccine candidates are shown in Table 1.

### 3.5. Challenges and Future Directions

Despite encouraging experimental data, significant challenges remain in developing and deploying WNV vaccines for birds. Commercial equine vaccines such as Duvaxyn^®^ and Recombitek^®^ have also been evaluated in raptors, including falcons. Three-dose vaccination regimens reduced clinical disease severity and viral shedding but did not provide complete sterilizing immunity. Notably, incomplete suppression of viremia following two doses raised concerns about residual transmission risk to mosquitoes. Testing of Duvaxyn^®^ in flamingos and red-tailed hawks failed to develop a humoral response after immunization with this vaccine [73]. Extensive necrotic lesions were documented in the pectoral muscle at the site of vaccine inoculation of western scrub-jays (*Aphelocoma californica*) vaccinated with the RECOMBITEK^®^ [75]. The canarypox-vectored vaccine expressing WNV prM/E proteins (marketed as Recombitek^®^ in the U.S. and Proteq WNV in Europe) showed slightly improved efficacy in reducing clinical signs and viral shedding. However, occasional adverse local inflammatory reactions limited its suitability for widespread avian application.

Beyond species-specific immune variability, economic constraints, logistical difficulties in vaccinating wild populations, storage conditions for certain vaccine types, and interference with serological surveillance all limit large-scale implementation. Oral vaccines have shown poor performance compared with intramuscular administration. Nevertheless, continued efforts to vaccinate high-risk and captive bird populations, especially potent amplifiers such as corvids and raptors, may play an important role in reducing viral amplification and spillovers to humans and horses.

**Table 1:** Bird WNV vaccine candidates evaluated in clinical trials.

Vaccine/Platform	Species	Quantitative Efficacy	References
DNA vaccine (prM-E-genes)	Crow	~40–60% survival (partial protection)	[76]
DNA vaccine (prM-E-genes)	American robin	~80–100% survival	[77]
Recombinant WNV E-protein subunit (WN-80E)	Domestic geese	~100% protection (no viremia)	[78]
Fowlpox/Canarypox-vectored WNV constructs	Domestic geese	~85–87% survival	[79]
Recombinant subviral particle (RSP) WNV vaccine	Eurasian magpies	~70–71% survival	[73]

## 4. Equine Vaccine Development, Clinical Trials, and Successful Vaccine

Like humans, WNV infection can cause serious disease in horses, most often involving the nervous system and leading to substantial illness and death. After infection, horses develop only low and short-lived viremia, which is not sufficient to infect mosquitoes. As a result, horses cannot contribute to continued virus transmission and are therefore considered incidental, dead-end hosts in the WNV transmission cycle. Despite this, horses play an important role in WNV surveillance. Increases in equine WNV cases often occur before or at the same time as outbreaks in humans, reflecting shared exposure to infected mosquitoes and similar environmental conditions. This close timing highlights the value of monitoring WNV infections in horses as an early warning system that links veterinary surveillance with public health efforts [80]. More recently, a notable increase in WNV activity was reported in Spain during 2021–2022, with higher case numbers, wider geographic spread, and more severe disease observed in both humans and horses. These observations raise concern that future WNV outbreaks may occur more frequently and affect broader regions, potentially driven by climate change, changes in land use, and the ongoing expansion of mosquito vectors capable of transmitting the virus across southern and western Europe [81].

At present, there are no approved antiviral therapies available for the treatment of WNV infection in either humans or horses, and clinical management remains largely limited to supportive and symptomatic care. The absence of targeted therapeutics underscores the importance of preventive approaches, particularly vaccination, which remains the most effective strategy to reduce the risk of neurological disease, decrease mortality, and limit the overall burden of WNV infection. As a result, sustained efforts have focused on the development and optimization of vaccines for equine populations, with the goal of preventing viremia and reducing the severity of clinical disease following infection [82]. These vaccination strategies have proven effective in lowering disease incidence in horses and serve as an important model for preventive approaches against WNV in the absence of licensed antiviral treatments.

Vaccination takes advantage of the immune system’s natural ability to generate long-lasting protection without causing active infection or disease [83]. After intramuscular injection, vaccine antigens are captured by dendritic cells, which become activated and migrate to nearby lymph nodes where immune responses are initiated [84]. In these secondary lymphoid organs, antigen-presenting cells stimulate naïve CD4^+^ and CD8^+^ T cells and support B cell activation, leading to antibody production and immune maturation [85]. Activated B cells undergo affinity maturation and immunoglobulin class switching, processes that improve antibody quality and antiviral function [86]. This coordinated response results in the rapid generation of short-lived plasma cells and the appearance of high affinity neutralizing antibodies, often detectable within one to two weeks following vaccination. Long-term protection is maintained by the formation of memory B cells and long-lived plasma cells that persist in the bone marrow and continue to produce protective antibodies over extended periods [87]. Together, these mechanisms allow vaccination to provide durable immunity while minimizing the risk of disease.

Optimal protection against West Nile virus in horses requires the coordinated engagement of both humoral and cellular arms of the immune system. To date, four vaccines have received approval for equine use in the United States by the U.S. Food and Drug Administration, three of which remain commercially available. Among these, the Recombitek Equine West Nile Virus Vaccine, developed and licensed in the United States, employs a recombinant canarypox virus vector engineered to express the WNV prM and envelope (E) proteins, allowing for robust activation of antibody-mediated immunity alongside virus-specific T cell responses. This platform has been shown to elicit strong neutralizing antibody titers as well as interferon-γ–producing T cells, illustrating the advantages of vaccine strategies that stimulate complementary immune pathways [88].

In contrast, inactivated whole-virus vaccines such as West Nile-Innovator (widely used in the United States and North America) and Vetera WNV (licensed in the United States and used in selected international markets) primarily confer protection through the induction of high-titer serum-neutralizing antibodies. These formulations provide protection for up to one year following booster immunization and are generally well tolerated, with a low incidence of adverse effects. Notably, West Nile-Innovator^®^ has demonstrated protective efficacy approaching 94% against clinical disease in field and experimental studies conducted in North America, underscoring the effectiveness of antibody-focused vaccine approaches in preventing severe outcomes [89].

The first FDA-approved DNA plasmid vaccine for equine WNV encoded the viral prM and E proteins and incorporated the metastim adjuvant to enhance immunogenicity. This vaccine successfully induced neutralizing antibody responses and detectable cell-mediated immunity, establishing proof-of-concept for nucleic acid-based vaccination in veterinary medicine [90]. However, high production costs, regulatory hurdles, limited market demand, and unresolved questions regarding long-term durability ultimately led to its withdrawal from commercial use. As a result, no DNA-based WNV vaccines are currently available for veterinary application, despite their demonstrated immunological potential [90].

Historically, much of WNV vaccine development has relied on empirical strategies centered on viral isolation, inactivation, and administration, often with limited integration of mechanistic immunological insight during early design stages. The consequences of this approach were exemplified by the withdrawal of the PreveNile^®^ vaccine in 2010, an inactivated yellow fever 17D-based chimeric vaccine that initially showed strong immunogenicity in pre-licensure studies. Post-marketing surveillance, however, revealed rare but severe adverse events, including acute anaphylaxis, colic, respiratory distress, and death, ultimately necessitating its recall. These outcomes exposed critical deficiencies in pre-clinical immune profiling and safety prediction, particularly in identifying vaccine-associated reactogenicity and immune-mediated toxicities before widespread use [91]. Equines’ WNV vaccine candidates evaluated in clinical trials are shown in Table 2.

## 5. Human Vaccine Development, Clinical Trials, and Successful Vaccine

Currently, extensive research utilizing several vaccine platforms including live attenuated, inactivated, DNA, and subunit-based approaches have advanced into clinical and preclinical evaluation. Vaccine development has been complicated by multiple biological and practical challenges, including the predominant risk in older adults, unpredictable outbreak patterns, limited commercial incentives, and the absence of a clearly established immune correlate of protection. Thus, despite spanning more than two decades, no licensed human vaccine is currently available for WNV, even though the virus continues to cause seasonal outbreaks and neuroinvasive disease worldwide. This section critically reviews major human WNV vaccine candidates (Table 3), summarizes clinical findings, and discusses key scientific and translational obstacles impeding their successful development and licensure.

### 5.1. Live Attenuated Vaccine Candidates

One of the two main live attenuated vaccines that have undergone clinical trials is the chimeric virus made from the yellow fever (YF) 17D vaccine virus as a vector in which the prM-E protein genes are replaced with prM-E sequence of a strain WN NY99 isolated from the brain of a flamingo with fatal encephalitis from New York in 1999. The first version was ChimeriVax-WN01, which was developed as a vaccine for use in horses. Further, a more attenuated vaccine candidate designated as ChimeriVax-WN02 was developed for human use by changing three amino acid changes in the E protein of ChimeriVax-WN01 at residues 107, 316 and 440 [93]. In phase I clinical trial, healthy adults aged 18–40 years administered with the lower dose of ChimeriVax-WN02 had statistically higher viremia than those receiving a 100 times higher dose [94]. Further, two phase II trials were performed focusing safety of the vaccine particularly in the older age group. The first phase II trial was divided into two parts and included adults aged 18–40 years (part 1, *n* = 112) or ≥41 years (part 2, *n* = 96) [95]. Approximately ∼96% of subjects in part 2 achieved seroconversion at day 28 of the active treatment. However, the occurrence of treatment-emergent adverse events (TEAEs) was higher in the higher age group treated with a 3.7 × 10^5^ PFU dose of ChimeriVax-WN02 than the lower-age subjects from part 1 study. In the second phase II clinical trials, a total of 480 subjects (120 subjects per group) provided immunogenicity data, particularly to identify common AEs [96]. Similar to the first phase II trial, the most common systemic AEs reported were general body symptoms such as headache and malaise, and also, the seroconversion rates were dose-dependent; however, the differences were not statistically significant. The neutralizing antibody titer was calculated through a PRNT50 and found to be increased by day 28 in the active vaccine groups, ranging between 600 and 688, but not in the placebo group (5.93). The neutralizing antibody titer was found to be a PRNT50 titer of ≥10, considered protective against other flavivirus diseases such as Japanese encephalitis and YF [97]. However, PRNT50 in this study used the ChimeriVax-WN02 vaccine strain as the challenge virus instead of the wild-type virus. The changes in antigenic domain II of the E protein with mutations at position 107 in other flaviviruses were shown to have less binding with monoclonal antibodies targeting the highly conserved fusion loop of E protein [98]. Therefore, it is premature to conclude that titers ≥ 10 are protective against WNV infection. In addition, the immune response kinetics were not recorded before and after 28 days of vaccination. Thus, more studies must be done to confirm the true protection conferred by the vaccine, as no antibody correlate of protection has been established.

The second live attenuated vaccine candidate “rWN/DEN4Δ30” was evaluated in three phase I clinical trials. In this chimeric vaccine, the prM and E structural protein genes of the DEN4Δ30 virus have been replaced by those of WNV NY99 [99]. Attenuation of the rWN/DEN4Δ30 virus was achieved by deleting the 30-nucleotide 3′ untranslated region (UTR) and by antigenic chimerization of WNV with non-neuroinvasive dengue virus serotype 4 (DENV-4). In the first two phase I trials including 82 (*n* = 82), subjects of age 19–50 years received a single or two doses of rWN/DEN4Δ30 of different pfu [100]. The third clinical phase I trial was performed with two doses of 10^4^ PFU rWN/DEN4Δ30 in 50–65 year-old adults (*n* = 28). The booster dose was given post-180 days of the first 0.5 mL subcutaneous injection. A single dose of rWN/DEN4Δ30 was able to achieve an acceptable balance of immunogenicity and attenuation [101]. However, the duration of this protection or neutralizing antibody from a single vaccination needs to be examined in further studies.

### 5.2. Safety Concerns with Live Attenuated Vaccines

Prolonged viremia and adverse events: Live attenuated vaccines for WNV are engineered to maintain immunogenicity while reducing pathogenicity. However, in populations most at risk such as older adults or individuals with pre-existing health conditions these vaccines pose a potential risk for prolonged viremia (extended circulation of the virus in the bloodstream). This increases the likelihood of adverse reactions, including systemic inflammation or severe illness. Although ADE is a well-documented concern for other flavivirus vaccines (e.g., dengue), it has not been conclusively observed with WNV vaccines. ADE occurs when non-neutralizing antibodies from previous flavivirus infections enhance subsequent viral entry into cells, potentially worsening the infection [102,103]. While WNV infections have not demonstrated this phenomenon, the theoretical risk remains a significant barrier to broader vaccine adoption and clinical trials.

### 5.3. Inactivated Vaccine Candidate

The first clinical trial of an inactivated WNV vaccine was based on a naturally attenuated strain, such as WNV Kunjin (WNV-KUNV). WNV-KUNV was first isolated in Australia in 1960 with no documented fatal human infection [104,105]. WNV KUNV is genetically similar to WNV New York (WNV-NY) (98% amino acid identity), which initiated the WNV epidemic in the United States. This might be one of the reasons behind the complete protection by the live and infectious WNV-KUNV strain against the lethal infection of North American WNV isolates in mice. Even H_2_O_2_-inactivated WNV-KUNV protected against lethal WNV challenge by inducing a robust polyfunctional antigen-specific CD8^+^ T cell response in human HLA-A2 transgenic and wild-type C57BL/6 and BALB/c adult mice [106]. This hydrogen peroxide-inactivated whole virion adjuvanted with aluminum hydroxide was termed as HydroVax-001 WNV.

In preclinical trial in monkeys, neutralizing antibody titers were analyzed against lineage 1 and lineage 2 WNV strains. Two months after a HydroVax-001 WNV booster dose, the amount of neutralizing antibodies was fairly consistent across the different lineage 1 strains, with geometric mean titers (GMTs) ranging from 190 to 453 compared to a low GMT range between 57 and 113 for lineage 2 strains [107]. Nevertheless, neutralizing antibody responses from HydroVax-001 WNV vaccination were comparable to a group of human subjects (*n* = 11) who had been infected with WNV during a 2003 outbreak in Colorado.

Given these promising results, a two-dose intramuscular (IM) injection was scheduled at a 28-day interval to assess safety and immunogenicity in phase I clinical trials in humans. At a 1 mcg dose, HydroVax-001 was not immunogenic by PRNT50 but elicited up to 41% seroconversion by WNV-specific ELISA after the second dose. At a 4 mcg dose and added complement, seroconversion rates increased to 50%, and 75% as observed by PRNT50 and WNV-specific ELISA, respectively [61]. HydroVax-001 performed averagely and was not efficient in eliciting durable neutralizing antibody responses against WNV. The clinical study suggests that the inactivated vaccine approach could be improved by the third dose of vaccine administration. In addition, an alternate inactivation approach can be employed to retain maximum neutralizing epitopes. One study described using an oxidation approach for the development of inactivated vaccines that utilizes reduced concentrations of H_2_O_2_ in combination with copper and methisazone (MZ). Further, the addition of formaldehyde provided a robust two-pronged approach that led to more efficient virus inactivation with improved retention of neutralizing epitopes [62].

### 5.4. Safety Concerns with Inactivated Vaccines

Inactivated WNV vaccines are considered safer than live attenuated vaccines because they are non-replicating and cannot revert to virulence, making them suitable for immunocompromised and elderly individuals [108]. However, chemical inactivation methods such as formaldehyde or hydrogen peroxide can alter viral protein structure, potentially reduce the quality of neutralizing antibody responses and raise theoretical concerns about immune enhancement [109]. The use of adjuvants like aluminum hydroxide improves immunogenicity but is associated with mild local and systemic reactogenicity [110]. In addition, incomplete viral inactivation remains a theoretical manufacturing risk, requiring stringent validation and quality control. Furthermore, repeated booster doses may increase cumulative reactogenicity and affect tolerability. Additionally, while candidates such as HydroVax-001 are generally well tolerated, their relatively modest and short-lived neutralizing antibody responses highlight the need for improved formulations that balance safety with durable immunogenicity [61].

### 5.5. DNA Vaccine Candidate

A total of two DNA vaccine candidates, VRC-WNVDNA017-00-VP and VRC–WNVDNA020-00-VP, have been tested in two separate clinical phase I trials. Both of these DNA vaccine candidates were closed, circular plasmids encoding prM and E proteins identical to those of the WN NY99 human isolate. In the first DNA vaccine candidate VRC-WNVDNA017-00-VP, a modified signal sequence from JEV was added upstream of the WNV prM and E coding sequences and cloned into the expression vector with a CMV promoter, whereas in the second DNA vaccine construct VRC–WNVDNA020-00-VP, a modified CMV/R promoter was constructed adding a regulatory sequence for the R region of the long terminal repeat from the HTLV-1, known to enhance the transcription and post-transcriptional events [111,112].

A total of 15 human subjects were tested for the phase I clinical trial (VRC 302), administering a 4 mg dose of DNA vaccine (VRC-WNVDNA017-00-VP) with a CMV promoter via intramuscular injection [111]. Vaccine-induced neutralizing antibodies were detected by ELISA at weeks 8, 12, and 32. At week 12, among 15 subjects, six, three, and one subject exhibited reciprocal antibody titers of 480, 1920, and 7680, respectively. Moreover, enhanced CD4^+^ T cell responses were elicited against the WNV-E-specific antigen and were more frequent than CD8^+^ T cell responses.

The second phase I trial (VRC303), evaluated a WNVDNA020-00-VP vaccine with a vector construct having the CMV/R promoter. The study included 30 healthy adults (*n* = 30) of age 18–50 years in one group and age 51–65 years in the second group [112]. Three doses of the vaccine regime were administered on study days 0, 28, and 56. The frequency and magnitude of T cell responses to the prM protein were significantly increased compared to the VRC302 trial. In earlier reports, DNA vaccines have been shown to elicit vaccine-specific antibodies and not been known to elicit neutralizing antibodies [113,114]. But, the VRC-WNVDNA017-00-VP and VRC–WNVDNA020-00-VP WNV DNA vaccines induced neutralizing antibody production, which might be possible due to the expression of prM and E gene products resulting in the formation of non-infectious virus-like particles called “subviral particles”. Secondly, the delivery of these DNA vaccines using Biojector might have enhanced augmentation in aged skin. Needle-free immunization has been shown to deposit antigen in a cone like distribution into the epidermis and dermis, directly to the sites of dendritic cells that migrate to draining lymph nodes after activation [115]. In these studies, neutralizing antibody peak responses were measured at around week 12 but remained positive at relatively lower magnitude by the end of the year-long study. Secondly, it is important to complete three-dose regimes to gain an effective long-term antibody response.

### 5.6. Safety Concerns with DNA Vaccines

DNA vaccines for WNV have shown a favorable safety profile in early-phase clinical trials, largely due to their non-infectious and non-replicating nature, eliminating risks of reversion or viral spread. However, several safety considerations have been discussed in the literature. A theoretical concern is the potential integration of plasmid DNA into the host genome, although this has not been observed at significant levels in clinical studies. Additionally, DNA vaccines can trigger innate immune activation, which may lead to mild local or systemic reactions such as injection-site pain or transient inflammation [111,116]. The use of delivery systems like needle-free injectors (e.g., Biojector) may further contribute to local tissue irritation [117]. Another concern is the requirement for multiple high-dose administrations (e.g., three-dose regimens), raising questions about long-term tolerability and cumulative immune stimulation. Despite these considerations, clinical trials (VRC302 and VRC303) reported that the vaccines were generally well tolerated, with no serious adverse events, supporting their overall safety while highlighting the need for continued monitoring in larger populations [112].

### 5.7. Subunit Vaccines

Subunit vaccine strategies for WNV mainly target the envelope (E) protein and its domain III (EDIII), as these regions contain important neutralizing epitopes. In preclinical studies, recombinant E protein vaccines produced in insect or mammalian systems have shown strong neutralizing antibody responses and protective efficacy in mice and non-human primates, although they typically require adjuvants such as aluminum salts or other immunostimulants to enhance their effectiveness [118,119,120]. EDIII-based vaccines, including recombinant and nanoparticle-linked forms, provide greater antigen specificity and may reduce non-neutralizing responses, but they often show lower immunogenicity when used alone [91]. Virus-like particle (VLP) vaccines composed of prM and E proteins represent a more advanced approach, as they closely resemble the native virus structure and induce both antibody and T cell responses in animal models [121].

### 5.8. Challenges with Subunit Vaccines and Advancement in Adjuvants and Immunoinformatics

Despite encouraging preclinical findings, the clinical development of WNV subunit vaccines has been limited. Only a few candidates have entered early-stage or phase I trials, where they have shown good safety but variable immune responses. Overall, improving antigen design, adjuvant use, and dosing strategies will be important to achieve stronger and longer-lasting protection in humans. Subunit vaccines are often not immunogenic and must be paired with an immune-stimulating adjuvant to improve efficacy. Recently, there has been interest in molecules that activate mast cells (MCs) as potential vaccine adjuvants. MCs are innate immune cells that contain intracellular secretory granules, which in turn are loaded with inflammatory mediators such as prostaglandins, histamine, cytokines, and leukotrienes [122]. MC activators (MCAs) result in enhanced adaptive immune responses through the production of protective antibodies, but not IgE antibodies [123,124]. Different MCAs encapsulated in acetylated dextran microparticles are combined with the WNV Envelope III protein (EDIII) and their vaccine adjuvant activities are compared in vivo. Some of the MCAs produced a robust antibody response against EDIII protein [125]. These results emphasized the advantages of exploring better microparticle delivery platforms for the poorly soluble MCAs.

When screening novel adjuvant technologies, scientists are utilizing state-of-the-art immunoinformatic techniques. B cell and T cell epitopes of immunogenic proteins are identified and docked with B cell receptors, MHC-I, and MHC-II through computational models. The prominent affinity against T cell epitope NAYYVMTVGTKTFLV is observed with MHC II during molecular docking simulation analysis; hence, it needs to be verified as a potential candidate for a WNV epitope-based vaccine in future studies [126]. Furthermore, multi-epitope-based vaccine designs integrated the selected epitopes via GPGPG linkers, and a Cholera Toxin B subunit adjuvant with the help of another linker EAAAK, to enhance immunogenicity [127].

### 5.9. Other Challanges to Effective WNV Vaccine Development

#### 5.9.1. Cost and Feasibility

Economic constraints of vaccine programs: WNV is geographically focal and outbreak-prone, with variable incidence rates. Cost-effectiveness models have questioned the economic viability of national vaccination campaigns, suggesting that a targeted approach focusing on high-incidence regions or at-risk age groups might be more practical. This strategy has been successfully applied in other vaccine programs, such as hepatitis A vaccination.

#### 5.9.2. Technology Transfer

Challenges in vaccine uptake: Beyond production costs, uptake of WNV vaccines could be limited by concerns over long-term immunity and the potential requirement for multiple booster doses. For example, veterinary WNV vaccines involve two primary doses and annual boosters, a model that could deter human use due to logistical and economic barriers. Difficulty in conducting phase III trials: The sporadic and unpredictable nature of WNV outbreaks complicates the design and implementation of large-scale efficacy trials. Identifying regions with consistent outbreaks and enrolling sufficient participants poses significant logistical hurdles. Moreover, evaluating endpoints such as the prevention of neuroinvasive disease or infection requires precise monitoring and differentiation between vaccine-induced and infection-induced immunity.

#### 5.9.3. Shift in Circulating WNV Lineage Strains

Currently, WNV isolates are classified into nine different genetic lineages, with lineages 1 and 2 being associated with severe (neurological) disease in humans [128]. While lineage 1 WNV strains were previously considered to be more pathogenic compared to lineage 2, the recent outbreaks in Europe have been the result of the explosive spread of neuroinvasive lineage 2 rather than lineage 1 isolates [129]. Some of the WNV vaccines, which are primarily based on lineage 1 strains, have demonstrated immunogenicity against diverse strains of the virus, but limited numbers of these have been studied against lineage 2 viruses [130]. These studies are confined to animal models and, thus, can provide relevant immune protection in animals, but the protection against lineage 2 virus in humans is yet to be confirmed. Thus, at present, there is no licensed human WNV vaccine available. One study examined the lineage 2 strains, namely SHUA-1 and SHUA-3, isolated from the blood and saliva respectively of the same patient diagnosed with neuroinvasive West Nile disease in Moscow, 2021, respectively [131]. The inactivated SHUA-3 strain provides 100% seroconversion and immunogenicity against SHUA-1 and 100% protection from morbidity and mortality in mice.

**Table 3:** Human WNV vaccine candidates evaluated in clinical trials.

Vaccine Candidate	Clinical Trial Phase	Study Population	Year	References
ChimeriVax-WN02	Phase I	Age 18–40 years, *n* = 80	2006	[94]
	Phase II	Age 18–40 years (part 1, *n* = 112); Age ≥ 41 years (part 2, *n* = 96)	2011	[95]
	Phase II	Age ≥ 50 years, *n* = 480	2012	[96]
rWN/DEN4Δ30	Phase I	Age 9–50 years, *n* = 82	2013	[100]
	Phase I	Age 50–65 years, *n* = 28	2016	[101]
HydroVax-001 WNV	Preclinical	Non-human primates	2017	[107]
	Phase I	Age 18–49 years, *n* = 51	2019	[61]
DNA Vaccine (VRC-WNVDNA017-00-VP)	Phase I	Age 18–50 years, *n* = 15	2007	[111]
DNA Vaccine (VRC–WNVDNA020-00-VP)	Phase I	Age 18–65 years, *n* = 30	2011	[112]

## 6. Conclusions

WNV continues to be an important public health threat, but developing an effective human vaccine has been challenging. Several vaccine platforms, including live attenuated, inactivated, DNA, subunit, and recombinant vaccines, have shown encouraging immune responses in animal models and early clinical trials. Despite this progress, each approach has important limitations. Key challenges include safety concerns in high-risk populations, incomplete understanding of immune protection, limited durability of immune responses, cross-reactivity with other flaviviruses, and the potential risk of antibody-dependent enhancement. Inactivated and DNA-based vaccines are generally safe but often require multiple doses or stronger adjuvants to produce long-lasting immunity. Live attenuated vaccines tend to induce stronger immune responses; however, their use raises safety concerns, especially in older adults and immunocompromised individuals. In addition to these biological challenges, vaccine development is further hindered by economic limitations, unpredictable outbreak patterns, and difficulties in conducting large-scale efficacy trials. Overcoming these obstacles will require improved vaccine designs, better adjuvant strategies, and carefully targeted vaccination approaches to support the development of a safe, effective, and practical human vaccine against WNV.

## Figures and Tables

**Table 2:** Equine WNV vaccine candidates evaluated in clinical trials.

S.No.	Vaccine Name	Viral Antigen	Licensed	References
1	West Nile-Innovator	Whole killed virus	YES	[89]
2	Vetera West Nile vaccine (Boehringer Ingelheim)	Killed virus	YES	[91]
3	West Nile-Innovator DNA (Discontinue)	Plasmid DNA prM/E	YES	[91]
4	Recombitek WNV	Recombinant canary pox vaccine	YES	[88,92]
5	PreveNile (Recalled)	WNV prm-E in yellow fever backbone	YES	[91]

## Data Availability

No new data were created or analyzed in this study.

## Abbreviations

ADE	Antibody-Dependent Enhancement
AES	Acute Encephalitis Syndrome
AFM	Acute Flaccid Myelitis
AFP	Acute Flaccid Paralysis
AE	Adverse Events
ALT	Alanine Aminotransferase
CD	Cluster of Differentiation
CMV	Cytomegalovirus
CNS	Central Nervous System
COPD	Chronic Obstructive Pulmonary Disease
CSF	Cerebrospinal Fluid
DC	Dendritic Cell
DC-SIGN	Dendritic Cell-Specific Intercellular Adhesion Molecule-3-Grabbing Non-Integrin
DENV	Dengue Virus
DNA	Deoxyribonucleic Acid
E	Envelope Protein
EAAAK	Glutamic Acid-Alanine-Alanine-Alanine-Lysine (Linker)
EDIII	Envelope Domain III
ELISA	Enzyme-Linked Immunosorbent Assay
ER	Endoplasmic Reticulum
FDA	Food and Drug Administration
GMT	Geometric Mean Titer
GPGPG	Glycine-Proline-Glycine-Proline-Glycine Linker
H_2_O_2_	Hydrogen Peroxide
HCA	Hierarchical Clustering Analysis
HLA	Human Leukocyte Antigen
HTLV-1	Human T Cell Leukemia Virus Type 1
IgE	Immunoglobulin E
IgG	Immunoglobulin G
IgM	Immunoglobulin M
IM	Intramuscular
JEV	Japanese Encephalitis Complex
MC	Mast Cell
MCA	Mast Cell Activator
MHC	Major Histocompatibility Complex
MZ	Methisazone
NS	Non-Structural Protein
PCR	Polymerase Chain Reaction
PFU	Plaque-Forming Units
PRNT	Plague Reduction Neutralization Test
PS	Phosphatidylserine
RGD	Arginine-Glycine-Aspartic Acid
RGE	Ariginine-Glycine-Glutamic Acid
RNA	Ribonucleic Acid
RT-PCR	Reverse Transcription Polymerase Chain Reaction
SAE	Serious Adverse Event
TAM	TYRO3-AXL-MERTK Receptor Family
TEAE	Treatment-Emergent Adverse Event
TIM	T Cell Immunoglobulin and Mucin-Domain
UTR	Untranslated Region
VLP	Virus-Like Particle
WNV	West Nile Virus
WNV-KUNV	West Nile Virus-Kunjin Stain
WNV-NY	West Nile Virus New York Strain
YF	Yellow Fever
YF-17D	Yellow Fever 17D Vaccine Strain
